# Parental views and the key role of nurses for high vaccine acceptance in Sweden – a focus group study

**DOI:** 10.1186/s12889-023-16678-5

**Published:** 2023-09-14

**Authors:** Emma Appelqvist, Madelene Danielsson, Asha Jama, Lina Schollin Ask, Christina Stenhammar, Ann Lindstrand, Kristian Riesbeck, Adam Roth

**Affiliations:** 1 Department of Public Health Analysis and Data Management, Public Health Agency of Sweden, Stockholm, Sweden; 2 Clinical Microbiology, Department of Translational Medicine, Faculty of Medicine, Lund University, Malmö, Sweden; 3 Department of Global Public Health, Karolinska Institutet, Stockholm, Sweden; 4 Department of Women and Child Health, Karolinska Institutet, Stockholm, Sweden; 5 Department of Immunization, Vaccines and Biologicals, Unit Essential Programme On Immunization, World Health Organization (WHO) Headquarters, Geneva, Switzerland

**Keywords:** Childhood vaccinations, National immunization program, Vaccine acceptance

## Abstract

**Background:**

In Sweden, vaccine uptake is exceptionally high due to an efficient child immunization program. More than 97% of Swedish children were vaccinated at child health care centers (CHCs) according to the schedule at 2 years of age in 2021. From the age of 6 years, vaccinations are given within the school health care. Maintaining high vaccination coverage over time is one of the central motives to explore and understand drivers for vaccine acceptance. The current study aimed to assess parental vaccine acceptance concerning the national immunization program and explore factors contributing to the high vaccine acceptance in Sweden.

**Methods:**

Parents of children aged 1–2 years and 8–12 years were recruited through purposive sampling and asked to participate in focus groups held in three cities in Sweden, in February and March 2019. In total, 47 parents participated in two focus groups per city, one session for parents of younger (1–2 years) and older (8–12 years) children respectively. The focus group discussions were analyzed using qualitative content analysis.

**Results:**

Parents of children aged 1–2 years expressed the themes; strong compliance to and protection of the value of vaccinations; parents feel safe with an attentive relationship with their nurse; the spectrum of communication needs is essential to meet.

For parents to children aged 8–12 years, the themes expressed were; vaccinate to do good for the individual and society; a foundation of trust is built at CHCs for decisions later on; decisions for vaccination become more complex as children get older; communication changes as children get older and need to be explicit and tailored to the situation.

**Conclusion:**

Both individual and societal perspectives were shown to influence the vaccination decision for childhood immunizations, as manifested in parental reflections and experiences. As nurses have a key role, it is important to provide them with continued support and tools to facilitate their support for parents in making informed decisions. Continuous work for supporting driving factors for vaccination over time is needed to maintain high vaccine acceptance in Sweden.

**Supplementary Information:**

The online version contains supplementary material available at 10.1186/s12889-023-16678-5.

## Background

In 2021, more than 97% of children were vaccinated according to their schedule at 2 years of age in Sweden [1]. In Sweden, the national immunization program (NIP) is offered voluntarily, free of charge, to all children and reaches all socioeconomic groups [2]. The foundation of the Swedish NIP is the local child health care centers (CHCs) responsible for all children aged 5 years and younger and 99% of Swedish children attend the services [2]. A trustful relationship is built between the child health nurse and the family during the first year including at least 9 general health checks during the first year, one of which is an at-home visit by the nurse [3]. During the child’s 5 years at the CHCs, 6 visits include vaccinations of which 4 visits take place during the first 12 months. As at least one caregiver brings the child to health checks, only oral consent is required for vaccination. For children 6–17 years of age, the school health care services take over the responsibility for implementing vaccinations in the NIP. During these years, children are offered vaccinations on 4 occasions [4]. In contrast to vaccinations offered at CHC, written consent to vaccinate has to be signed by the caregivers for vaccinations in schools. At the time of our study, the overall NIP included 10 vaccinations [4]. Exploring and understanding factors for parental vaccine acceptance and periodic monitoring of those factors in the Swedish context is important to support and promote a resilient NIP over time.

Vaccine acceptance is a broad term that has been described to include the extent to which individuals accept, question or refuse vaccination [5] whereas more recently it has been defined to include the decisions to either accept, or decline vaccination when offered an opportunity to vaccinate [6, 7].

This decision is influenced by various factors and may therefore potentially change according to the situation and context. The World Health Organization (WHO) emphasizes the importance of monitoring behavioral and social drivers (BeSDs) for vaccine uptake [8]. The vaccine acceptance relates closely to behavioral and social drivers (BeSD) of vaccination, which include emotional, psychological, motivational and practical aspects [9]. The SAGE Working Group on Vaccine Hesitancy defined vaccine hesitancy as a “delay in acceptance or refusal of vaccination despite the availability of vaccination services” [10]. The factors influencing vaccine acceptance and the behavioral and social drivers for vaccination are complex, dynamic and specific to different contexts.

Studies in Sweden have highlighted the role of health care professionals as the most trusted source for parents on information regarding childhood vaccinations [11, 12]. Qualitative studies have highlighted the complexity of parental decision-making for HPV vaccination for girls [13, 14]. The importance of robust organizations for the implementation of childhood vaccinations has been highlighted previously where the CHCs and the school health care were essential settings for high and equitable uptake [15, 16]. Previous quantitative and qualitative studies conducted in Sweden have revealed parental concerns on vaccine safety of vaccinations included in the NIP even though the parents decided to vaccinate their children [11–14]. Previous results suggest that about 20 percent of parents vaccinated their child but had questions and concerns while vaccinations against MMR and HPV were the most frequently postponed vaccinations due to parents’ concerns about the safety of the vaccines [11]. In 2019, the year of the current study, the HPV vaccination coverage for girls born in 2006 and 2007 was 86 percent for the first dose [17].

Understanding drivers for vaccine acceptance is a central aspect to support and maintain the exceptionally high and stable vaccination coverage for childhood vaccinations within the NIP over time. The Swedish Public Health Agency has an overarching mission for the government to support and monitor the NIP, including sustaining its resilience and parental vaccine acceptance. An additional government assignment to strengthen children’s protection against communicable diseases provided the means to explore key issues relevant for long-term vaccine acceptance [18]. This study aimed to assess parental vaccine acceptance for vaccinations in the NIP and explore factors contributing to the high vaccine acceptance in Sweden.

## Methods

### Recruitment of participants

Parents to children aged 1–2 years and 8–12 years were invited to participate in focus group discussions (FGDs). The age ranges of the children were chosen based on the NIP schedule for having been offered recent vaccinations. Children aged 1–2 years had been offered vaccinations at CHCs and those aged 8–12 years had been offered NIP vaccinations, including HPV vaccination for girls, in school health services.

Participants were recruited through purposive sampling to include various perspectives, views and experiences among the parents. Recruitment was conducted by using a phone registry to call parents of children in the selected age ranges. During the phone call, the scope of the study was presented briefly to the parent. Eligible parents were recruited to ensure variance in sex, educational level, income and country of birth (born in Sweden versus abroad). To participate, the parents have had to be involved in the decision-making process for their children´s vaccinations, and all parents were required to have accepted vaccination for their child at least once within the NIP to be included in the study. The inclusion criteria were assessed during the recruitment call, and emphasis was also placed on voluntary participation in the study.

### Design of guide

The FGD guide (see supplemental file) covered topics including experiences of childhood vaccinations generally, vaccine preventable diseases (VPDs), experiences of vaccination appointments for children, decision-making as a parent, information for informed decisions and trust in information sources. For the older children, additional questions included experiences with HPV vaccination.

An external consultant with extensive experience in conducting FGDs was informed by the research team about the NIP and vaccine acceptance before conducting the FGDs. After the first FGD, the research team and the external consultant discussed the guide to assess the potential needs for improvements before it was finalized. However, no changes were done, and the same FGD guide was thereafter used in all FGDs.

### Process for data collection

Six FGDs were conducted in the cities of Malmö (metropolitan city), Stockholm (capital city) and Sundsvall (urban city) between February 25^th^ and March 4^th^ 2019. The locations of the FGDs had a geographical spread and included the southern (Götaland), middle (Svealand) and northern (Norrland) part of the country. In each city, one FGD was held with parents to children aged 1–2 years and 8–12 years, respectively. Written informed consent to participate in the study was obtained from all participants before the start of the FGDs. The study was approved by the Swedish Ethical Review Authority (Dnr 2019–00122). Data collected was anonymized to ensure that no individual could be identified. The number of participants varied from 4 to 11 in the 6 FGDs. In total, 47 parents participated of which 22 participants (18 mothers and 4 fathers) were parents of children aged 1–2 years and 25 were parents (13 mothers and 12 fathers) of children aged 8–12 years. The 6 FGDs ranged between 88 and 116 min with an average of 108 min.

### Data analyses

All FGDs were audio recorded and transcribed verbatim. The FGDs were thereafter analyzed using content analysis [19]. The method of content analysis was chosen to interpret and understand both the manifest as well as the more latent meaning of the FGDs while keeping close to the view of the participants. Recordings were listened to and transcripts were read multiple times for a thorough understanding of the material. The analysis of the FGDs of the two different age groups of children was conducted separately as the services for vaccinations differ. All transcripts were coded (EA). Codes were then assessed for similarities and differences and sorted to generate subcategories and categories. Lastly, themes emerged to highlight the latent findings. Throughout the analytical process, the research team frequently met to discuss interpretations of subcategories, categories, and themes. Following discussions and reflections, subsequent revisions of the results were made until the final results were agreed upon. Analyses were conducted in Microsoft Excel.

## Results

### Results from FGDs with parents of children aged 1–2 years

Three different themes emerged from the FGDs with parents of children aged 1–2 years (Table 1). In the text below, the themes are presented as headings and the categories as italics. Descriptive quotes from the FGDs are presented to support the context.

**Table 1:** Overall results from FGDs with parents of children aged 1–2 years

**Theme**	**Category**
**Strong compliance to and protection of the value of vaccinations**	Trust in the national immunization program
	Feelings of safety and solidarity motivate vaccination
**Parents feel safe with an attentive relationship with their nurse**	Diversity in how nurses encounter parents
	Need for responsive and understanding dialogue
	Practical vaccination skills are valued by parents
**The spectrum of communication needs is essential to meet**	Different needs of content, amount, and timing of information to feel prepared for a vaccination offer
	Diverse information channels and formats are needed
	Risk perception and sense of disease severity for vaccine preventable diseases

#### Theme 1. Strong compliance to and protection of the value of vaccinations

Trust in vaccines, the program and a norm to vaccinate were relevant factors, as well as feeling safe with the decision. Parents described vaccinations as something they just do automatically, both relying on a general norm and feeling confident and *trust in the national immunization program* and the child health care services involved in the NIP.

“for me it was just get to follow with the program”

“I also trust there is a reason for implementing a large national program”.

Parents described that *feelings of safety and solidarity motivate the vaccination*, underpinned by the comfort of knowing that their child has protection against diseases when being vaccinated. The solidarity beliefs included both a global perspective and reflections on herd immunity, as in protecting the most vulnerable in society by vaccination. Strong emotions emerged when discussing non-vaccinating parents, as they were perceived as not showing solidarity. Parents expressed frustration against non-vaccinating parents by explaining the consequences on an individual and societal level, as the third quote shows.

“just like a child car seat, it is [vaccinations] the safest option in most cases”

“It’s not just about oneself, there are others who cannot vaccinate their children [for medical reasons]”

“putting others at risk and causing large costs for the society”

#### Theme 2; parents feel safe with an attentive relationship with their nurse

In the second theme, a trustful relationship with the child health nurse was highlighted for parents’ decision-making. Diverse ways of interactions between nurses and parents were described.

Participants described *diversity in how nurses encounter parents* as they had different experiences of how nurses at the CHC offered vaccinations. Examples included perceiving the nurse to tiptoe around the topic of vaccinations, while others described the nurses to bring up vaccination calmly or in a direct manner. Another example given was that nurses just assumed that parents wanted to vaccinate.

“it was just it’s time for a shot”

“I realized that the encounter at CHC has really facilitated [vaccinations]”

Parents who had a positive encounter with their nurse also felt supported by a trusting relationship with the nurse that made them feel safe. Negative experiences on the other hand did not foster a trusting relationship. Such examples included feeling questioned by the nurse or experiencing a lack of trust. Flexibility in the information offered such as balanced information of adverse events and common reactions of the administered vaccine was described as important for parental vaccine decisions. Also, possibility to get in contact with the nurse and flexibility in the vaccination schedule for travels were highly appreciated.

“[focused] more on reactions and not the vaccine itself and its purpose”

“they described that it’s [fever reactions] normal, and to get touch with them [the CHC] if anything else showed up that would make me worried, it was calming to know [beforehand]”

Parents described a *need for responsive and understanding dialogue*. Experiences differed regarding the information received and the dialogues held with the nurses. On one end, parents were content while others were dissatisfied and felt a lack of attentiveness in the dialogue with the nurses and questions posed instead of the nurse being attuned to their specific needs as parents.

“much depends on the CHC-nurse, if the nurse is good you get good information and if she is not knowledgeable you don’t get any information”

Parents who had questions or concerns regarding vaccinations before making decisions for their child gave different examples, from the perception of the vaccines being new, wondering what the vaccines contain, and rumors of potential adverse events. Others were concerned in a more general sense such as worried to make the wrong decision. Examples were also given in which parents felt that the nurses acted strangely when they had concerns or debated whether to vaccinate or not.

“nervous for new things when it hasn’t been completely tested and you don’t really know”

“received information different sources, so I felt insecure and scared”

Parents expressed that the actual situation of when the child is being vaccinated can be challenging as it can be painful for the child and emotionally charged for the parents and thus *practical vaccination skills were valued by parents*. The practical skills of the nurses in offering pain relief strategies were highly valued and praised by the parents. Knowing that the nurses kept track of the vaccination record, but also made it easily accessible by writing in a booklet given to the parents, was perceived as a sense of safety by the parents.

“the nurse at CHC have an important role for the reaction of the child when getting the shot”

“explained explicitly and two nurses gave a shot in each leg, which felt safe”

#### Theme 3; The spectrum of communication needs is essential to meet

In the last theme, a wide spectrum of the need and timing of information about the vaccines was highlighted while information channels and the perception of the severity of the vaccine preventable diseases were also described.

Parents expressed *different needs of content, amount, and timing of information to feel prepared for a vaccination offer*. Parents suggested providing a plethora of information so that there is something for everyone and parents then can pick and choose what information to take part in.

“offer all the information there is and then you can [choose to] accept or decline”

Different perspectives were raised, on one side parents did not want much information at all whereas others requested in-depth information and scientific details. Also, the preferred timing of information differed as parents wanted information early during pregnancy whereas others were pleased with the current way at the CHCs.

“didn’t get much information at CHC but we also did not want information as we knew already [our decision to vaccinate]

“[suggestion to] provide a basis [of information] regarding vaccinations at prenatal check-ups”

Parents described *a need for diverse information channels and formats*. Of parents searching online, the “1177.se” the official Swedish health care information website was frequently used as a trusted source of information [20]. Other examples of information channels were family, friends, or online communication with other parents to share experiences and to get advice from others about child health. Preferences for how to access information also differed, *i.e.*, digitally, paper-based, or orally.

“especially when having the first child, I talked a lot with other parents regarding child health”

“I try to only use 1177, otherwise there is too much [information] out there and you need a PhD to assess what’s true or not”

In addition, *risk perception and sense of disease severity for the vaccine preventable diseases* were also highlighted. Participants described the vaccine preventable diseases included in the NIP as potentially life-threatening and were aware of serious following complications of infections. Reflections also included relatives that had experienced polio and measles.

“I remember the diseases somewhat but not exactly”

### Results from FGDs with parents of children aged 8–12 years

The results for parents to children aged 8–12 years revealed four different themes (Table 2). Descriptive quotes are shown below to support the context.

**Table 2:** Overall results from FGDs with parents of children aged 8–12 years

**Theme**	**Category**
**Vaccinate to do good for the individual and society**	Contribution to the community to protect the health of the individual and others
	Protect against serious diseases
	“We against them” mentality
**A foundation of trust is built at CHC for decisions later on**	Safety and trust in NIP and CHC
**Decisions for vaccination become more complex as children get older**	Vaccinations concerns shift as the child gets older and play a central role in the decision for HPV vaccination
	Challenges with vaccinations and worries for the future, although expressing positive aspects for vaccinations overall
**Communication changes as children get older and need to be explicit and tailored to the situation**	Need for transparent information for everyone
	Parents prefer and relate differently to information sources
	The child is a primary transmitter of information, especially for HPV vaccination

#### Theme 1; Vaccinate to do good for the individual and society

The first theme reflects the value of vaccination in various ways, both from a broader societal perspective and also protects against disease on the individual level. Parents described childhood vaccinations as a *contribution to the community to protect the health of the individual and others*. The childhood vaccinations and the program being offered free of charge were seen as a health-promoting measure for the population. The emphasis on solidarity was striking as parents expressed the need for vaccination for the collective good, and not only for their own child’s health but also for others, to protect vulnerable individuals. Parents just went along following the recommendations (passive decision) and accepted without questioning, reflecting the societal norm to vaccinate.

“won’t be a carrier of disease and put others who are weak at risk…even though my child has a good immune system and can get through the struggle of going through a disease”

Vaccinations were perceived to keep children healthy and *protected against serious diseases* and suffering, including cancer. Diseases were described as serious and potentially life-threatening events but parents struggled with details. Examples were given from a historic context, of people having died or of older relatives having personal experiences themselves.

“you avoid a lot of suffering, even for diseases that are not super dangerous, and the children don’t have to be sick”

The participants also expressed a critical view of parents who chose not to vaccinate their children, in line with the solidarity beliefs of vaccinating children. Examples were given where parents expressed that they believed the group of unvaccinated children was increasing in Sweden, a belief that seemed to trigger a *“we against them” mentality*. Views of how nurses should encounter non-vaccinators differed. Discussions with nurses were suggested as an essential element whereas, on the other hand, voices were also raised that discussions would rather just be detrimental and make more parents question and decline vaccinations.

“as more and more choose not to vaccinate… and as [my child becomes] adults they can become sick anyways as their own protection from vaccination decline”

“don’t remember what those opposed [to vaccinations] say but the facts they provide are not correct”

#### Theme 2; A foundation of trust is built at CHC for decisions later on

A core aspect of the second theme is the trust in vaccinations and the system which is built early on from the start of the NIP. Parents expressed *safety and trust in NIP and CHC*. Parents expressed gratitude for the service offered to their children and felt privileged. Parents recalled the experience of vaccination in the CHC as positive.

“felt straight forward at CHC, just to go along [and get vaccinated]”

“when the children were younger, it was easier to make decisions and I felt safe making them and could relate to it in a positive way”

#### Theme 3. Decisions for vaccination become more complex as children get older

Despite parents perceiving that the positive aspects of vaccination outweigh the negative, theme 3 reflects the challenges regarding the vaccine communication process that arise as children get older.

Parents perceived that *vaccination concerns shift as the child gets older and play a central role in the decision for HPV vaccination* **.** Parents perceived the new need for written consent rather than the previous oral consent at the CHCs to shift the decision to be more active. Also, vaccinations were perceived to be voluntary in schools and mandatory at CHCs. Moreover, there were more questions in general expressed for the school-age vaccines compared to the early childhood vaccines.

“I believe it was more difficult to make decisions regarding the vaccinations offered in school”

The HPV vaccination for girls was brought up as an example of generating many questions and concerns, making the decision of that vaccine more difficult than of others. Also, another aspect was the questions and concerns of the daughters themselves to be vaccinated against HPV infection but also acknowledging their central role for involving them in the decision-making process, which could be problematic at times.

“as children become older and part [of the decision] too, they have questions and concerns themselves…more questions makes it [the decision] more difficult”

“we had long discussions with my daughter as she wanted to understand and be included in the process [for HPV vaccination]”

Parents also expressed *challenges with vaccinations and worries for the future, although expressing positive aspects for vaccinations overall.* Examples of challenges with vaccines to school-aged children were the fear and temporary pain of needles and emotional aspects of the vaccination itself as well as parental fear of potential future adverse events in terms of “what if”. There was a general feeling of uncertainty that unexpected side effects might be revealed in the future years from now, specifically regarding the HPV-vaccinations. Parents also described a struggle with thinking of their daughter in a sexually active context, thinking their daughters were too young for talking about HPV-vaccinations.

“(the pain) is difficult for the child, but one can provide comfort”

“A vaccine can be good at the moment and one can read about it [about vaccine safety], but once you take the vaccination, what will happen in a few years time?”

Other worries, questions and concerns were also raised in a more general sense. Examples included the financial aspect of vaccinations and questioning the financial incentives in vaccination research and NIP. Despite the questions and worries, the benefits of vaccination outweighed the concerns for parents to vaccinate their children.

“my strongest reason not to vaccine was that I perceived it [vaccinations] to be very commercialized, not that we saw any particular risks [of vaccinating]”

#### Theme 4; Communication changes as children get older and need to be explicit and tailored to the situation

The fourth and final theme highlights the role of the school-aged child in the communication on vaccinations and the need for tailored and transparent information. Parents highlighted a *need for transparent information for everyone*. Moreover, parents requested transparent and objective scientific evidence, including pros and cons regarding vaccinations, such as possible adverse events and current knowledge gaps, and not just focusing on the benefits. Preference differed regarding the amount of information wanted as well as on timing and how to receive the information, from paper-based brochures to digital or oral communication.

“transparent information, including what is not known,.. communicated in a good, broad way [for all]…so that I can choose myself “

Parents *preferred and related differently to information sources* when searching for additional information other than the nurse at vaccination services. Searching online was common, however, voices were raised about the difficulty to assess the trustworthiness of the information. Talking to friends, family, parents or communicating in chats/forums online and exchanging experiences was also brought up, either to just get reassurance on their decisions or to discuss child health in a broader context.

“[talked to friends] to get reassurance in decisions or discuss something they’ve heard, they provide support in some way”

“I can google when I don’t find what I’m looking for, but then I have to be careful [in trusting information]”

At school, the *child was a primary transmitter of information, especially for HPV vaccination*, they were often the main information channel between the school and the parents. Parents received written vaccination information instead of information directly from the vaccinating nurse. On one end, parents wished to have received information themselves first, instead of receiving information from their child. Parents asked for oral information and the possibility to meet and discuss vaccinations with the nurse as well as the parents of the child’s classmates.

“just got a paper sent home, not so much information”

“just got informed that it [vaccination] would take place…the school was just the transmitter of information and what would happen regarding vaccination in grade 5”

“My perceptions is that the school is not like the CHC where they beforehand provided good information and care in a different way”

## Discussion

This study showed a strong parental trust in childhood vaccinations, from both individual and societal perspectives. Parents described that they wanted to vaccinate for the sake of their own children but also for others. The vaccinating nurse has a crucial role in vaccine acceptance in the Swedish context. The trust that is built with the nurse at the CHC when vaccinating in early childhood, built trust in the system and a strong foundation for the parents for later decisions of vaccinating school-aged children. Regardless of the child’s age, the respondents had a spectrum of communication needs that has to be met and tailored to a variety of preferences.

Previous studies have similarly highlighted the role of nurses and health care professionals, the importance of trust [21, 22] and the importance to meet the parents where they are at in their acceptance of vaccinations [23] as crucial factors for parental decisions regarding vaccinations. The complexities of parental decision-making for vaccinations have also been highlighted previously [22]. In this study, parents of younger children emphasized the importance of responsive and understanding dialogue, to feel seen and heard by their nurse. The responsiveness in understanding the parent and being able to listen and answer questions was highly valued. The described trusting relationship between nurses and parents also reflects the positive view of the quality of the services offered at CHCs. For parents of younger children, a spectrum of information needs was reflected, but sometimes information was not asked for or wanted, but rather the comfort of just going along with vaccination as a well-trusted system was described.

Parents perceived a more complex process of decision-making for vaccinations offered in schools compared to vaccinations offered earlier in childhood. Particularly for the decision of HPV-vaccination, it was evident that the child has a central role in contrast to when they were vaccinated as toddlers. Parents wanted to involve their child in the discussion and decision-making process. Similarly, another study found that parents and adolescents discussed vaccination decision-making but also as they matured, the adolescents got increasingly involved [24]. Parents with concerns about vaccination, in particular HPV, had a general feeling of uncertainty about “what if” adverse events that could show up in the future. Trusting the safety of HPV vaccination and the importance to vaccinate for the benefit of society highlighted in our current study has also been seen previously [14].

During early childhood, the parents have a direct relationship with the nurse at CHCs but in the school, there is a shift to the children to become the primary transmitter of information about vaccinations. It is therefore crucial to offer information to meet the variety of information needs and provide satisfactory and trustworthy information to limit the lack of information as a barrier to vaccination. Insufficient or dissatisfactory information along with trustworthiness in content, transparency and source of information influence decision-making [21, 22, 25, 26]. Especially in the perspective of HPV-vaccinations and also in the light of children’s right to information and participation in decisions [27], information regarding HPV vaccination needs to be provided also for children, tailored for their age. Information material targeting both parents and children can also be important to facilitate their discussions regarding vaccinations at home.

In our study parents of the older children expressed a “we against them” mentality, reflecting a critical view of parents who do not vaccinate their children. This salient view is potentially problematic, as increased polarization of the vaccination field may complicate the building of societal trust in vaccinations. Psychological studies have suggested that vaccinated individuals are not as generous towards non-vaccinated individuals [28]. In the social contract, it appears strongly that getting vaccinated is the morally right choice to do [28]. Themes for parents to both younger and older children in our study related to the social norm in terms of the compliance and value of vaccinations as well as the perspective of wanting to do good for society. The importance of the social norm to vaccinate should not be underestimated. Other studies have also emphasized the importance of having a pro-vaccine social norm and perception of public benefits for parents to vaccinate [22, 26].

A strength of using FGDs in our study design was that parents were given the opportunity to share thoughts and experiences in their own views and words. In addition, the purposive sampling of including parents with variances in background variables was done to capture different perspectives of parents. Throughout the analysis, the interdisciplinary research team has been an asset in increasing the trustworthiness of the study. Throughout the analysis from coding to generating themes, discussions among the team have been important to strengthen validity. Our study only included participants who had decided to vaccinate their children at least once. This was a deliberate choice as the large majority of parents in Sweden choose to vaccinate their children, and the aim was to better understand these parents. Other studies are needed to understand the perspective and experiences of adolescents and non-vaccinating parents and their perspective and experiences. Quantitative studies would be necessary to assess the magnitude and importance of various factors for vaccine acceptance. In addition, future studies should also assess the characteristics of the parents for vaccine acceptance. Our study did not include characteristics of the participating parents.

Although the school-based platform for the implementation of the NIP can be a key driver for HPV-vaccine acceptance [24], not only parental views and acceptance are the basis for vaccine uptake. Except for interpersonal and personal levels, the organizational level, as well as the community level and policies, have to be considered, as seen in a Canadian study [29]. As the vaccination coverage is high in Sweden, this study intended to understand those who accept vaccination. For the NIP, a deeper understanding of parents who choose to vaccinate and the factors contributing to vaccine acceptance is important in terms of guiding the process of building continued support and resilience in the national immunization program. Thus, the results of this study may contribute to improved support for nurses at CHCs and school health services and also for strengthening the communication regarding vaccinations for parents and children, specifically in regards to dialogue based communication strategies and tools for information and providing correct facts. It is important to keep providing support for vaccinating health care professionals and also parents for making informed decisions. The trust in vaccinations cannot be taken for granted, the situation can change quickly as seen in other countries with drastic drops in HPV coverage due to concerns in a short period of 2014–2015 [30, 31], however this was not seen in Sweden.

## Conclusion

To keep vaccine acceptance high, parents need to feel safe to make informed decisions, receive sufficient information and get their questions or concerns addressed. As children become older and may participate in vaccination decision-making as adolescents, they should receive age-appropriate support and information regarding vaccinations. Supporting the child-parent dialogue is equally important and thus, parents needs to be included in the communication process and be given resources for responding to upcoming questions also when the children are older, A continued adherence to the supportive societal norm of vaccination without contributing to the polarization of the vaccination field, building trust in vaccinations and especially in the nurses that inform and administer the vaccinations seem crucial for maintaining resilience in the NIP in Sweden. It is therefore important to assess parental vaccine acceptance and knowledge repeatedly and analyze what can be useful in supporting the implementation of the NIP. Insights gained will be important to guide strategies supporting parental vaccine decisions.

Both individual and societal perspectives were shown to influence the vaccination decision for childhood immunizations, as manifested in parental reflections and experiences. As nurses have a key role, it is important to provide them with continued support and tools as they in turn support parents to make informed decisions. Continuous work for tracking, understanding and cultivating driving factors for vaccination over time is needed to keep high vaccine acceptance in Sweden and to maintain and strengthen the resilience of the national immunization program for the future.

### Supplementary information

**Additional file 1.** Guide – Focus group discussions regarding childhood vaccinations.

## Data Availability

The datasets used and/or analysed during the current study available from the corresponding author on reasonable request.
