# Supplementary material for: Parental views and the key role of nurses for high vaccine acceptance in Sweden – a focus group study
Source: BMC Public Health. 2023 Sep 14;23:1786. doi: 10.1186/s12889-023-16678-5 (PMC10500778; doi:10.1186/s12889-023-16678-5)
Supplement: Supplementary file 1 — Additional file 1. Guide – Focus group discussions regarding childhood vaccinations. [file 12889_2023_16678_MOESM1_ESM.docx]

**Guide – Focus group discussions regarding childhood vaccinations**

**Background and aim**

Thank you for participating in our focus group discussion about parents’ experiences regarding childhood vaccinations. The Public Health Agency of Sweden is interested in understanding how you as a parent reason regarding vaccinations for children and the experiences you have from vaccinations of your child at child health care centers and school health services. Your experiences of information and communication regarding childhood vaccinations will also be discussed.

Participation in the focus group discussion is voluntary and anonymous.

The discussion will be recorded and documented by notes. The material from this session will be used for research. Therefore, we need your written informed consent to participate in this study (see separate document). The result of the study will be published in a report and also as a scientific article. The result will contribute to improving the information to parents regarding vaccinations.

**The structure for the FGD:**

- Participants area sked to sign written informed consent as they arrive (documents placed in the table for each chair).
- **Hello and welcome**. Presentation of hosts.
- **Brief background and aims** of the focus group, see separate document.
  - Clarify: informed consent, research, and gift card as thank you for participating.
- **Introduction to focus group discussion and the how it will be conducted**:
  A focus group discussion is a qualitative method that is suitable to use when aiming to understand attitudes in-depth – what individuals think, reason and why. As a group you represent parents to children aged 1-2 years/8-12 years but as an individual it is your experiences that are important, you don’t need to represent anyone else. Share as much as you can of your experiences. Listen and participate actively, let everyone finish with what they have to say, nothing is right or wrong, your experiences and views are equally important. What you say in this session is anonymous, your name will not be used. Please, put away any phones or computers during this session.
- **Time and breaks**. The discussion will last approximately 2 hours and as a thank you for participating you will recieve a gift card valued 500 SE at “[SuperPresentkort](http://www.presentkorttorget.se/)”. The gift card will be sent by email within a week of this session.
- **Roles.** Tina will guide you through questions while Jenny will be taking notes but might also ask questions if clarifications are needed. There will be one theme question that will be in focus followed by additional questions.
- **Round of presentation to warm-up:** Please tell us the age and sex of your child/children and briefly about the last time your child got vaccinated.

**FGD guide:**

1. **Theme question – spontaneous responses of vaccinations for children** *What are your experiences regarding vaccinations for children?*

Think freely, your spontaneous thoughts and feelings regarding vaccinations for children. Write down your thoughts on post-it notes, nothing is right or wrong. Write one note per though (max 5 post-it notes/person). Please add a happy, sad or neutral smiley depending on your attitude to vaccinations for children. Each and everyone will get to share what you have written and place the post-it note on the whiteboard. Please listen attentively when others are talking. The post-it notes will be categorized. We will have a dialogue of the categorization of the post-it notes on the whiteboard.

Depending on what was brought up in the theme question above, follow-up with relevant questions below:

### The diseases that are prevented by vaccinations in the national immunization program for children – what do you know about them?

*How sick do you get? Any particular diseases?*

### Why do we have vaccinations?

### Are vaccinations needed?

### *What is positive about vaccinations?*

### *Any particular vaccinations?*

### *How come you have these thoughts/feelings?*

### What do you know about childhood vaccinations?

### *Please describe.*

### For parents to children aged 8-12 years: Ask specifically about HPV vaccination, if it hasn’t been brought up previously.

### What is your experience of having your child vaccinated and the related decision-making?

*What are your experiences of being offered vaccinations for your child?*

*At the actual appointments, what did you think of it, what happened? (for instance how was the encounter by the nurse). Please share your stories and experiences.*

*Anything in particular that was positive or negative?*

*Any particular situation that you have in mind?*

*When and how did you make a decision?*

*How do you keep track of the vaccinations?*

### Information in relation to vaccinations

*Have you received any information? (before, at, or after the vaccination appointment)*

*How did you perceive the information?*

*Did you get your questions answered?*

*What could have been improved?*

*Did you actively seek information? What did you do?*

*Did you get answers to your questions?*

*When do you prefer to receive information? What information would you prefer?*

*In which instances would you want to receive information?*

*Any particular time you are more available to receive information?*

*How should the information be designed to be appealing to you?*

***Who do you trust as source for information/facts about vaccinations for children?****Do you talk to family and friends regarding vaccinations? Can you give/share some examples?*

*Have you heard or read anything striking about vaccinations that you remember in particular?*

1. **Final words?**

*At last – is there anything that yet hasn’t been discussed and that you would like to share or address?*

**Thank you!**
